# Sociodemographic Inequalities in Physical Activity in Latin America: Time for Policies Targeted at Groups that Need it the Most

**DOI:** 10.3389/ijph.2022.1605125

**Published:** 2022-11-10

**Authors:** André O. Werneck, Luciana L. Barboza, Ellen C. M. Silva, Raphael H. O. Araujo

**Affiliations:** ^1^ Center for Epidemiological Research in Nutrition and Health, Department of Nutrition, School of Public Health, University of São Paulo (USP), São Paulo, Brazil; ^2^ Postgraduate Program in Physical Education, University of Brasília (UnB), Brasília, Brazil; ^3^ Graduation Program in Health Sciences, Londrina State University, Londrina, Brazil

**Keywords:** public health, intervention strategies, exercise, physical activity, inequalities

**The IJPH series “Young Researcher Editorial” is a training project of the Swiss School of Public Health.**

Despite the benefits of physical activity for different health outcomes, many people are still not as active as the World Health Organization guidelines recommend. Global prevalence of insufficient inactivity (less than 150 min/week of moderate to vigorous physical activity) is 27.5% among adults [1]. Prevalence is higher in Latin America and the Caribbean, where 39.1% of adults and 81.1% of adolescents are insufficiently physically active [1, 2]. Women and people of lower socioeconomic status are even more likely to be insufficiently active, especially during leisure-time, adding to health inequality.

People are physically active at different times and under different circumstances, e.g., during leisure-time, transportation, work, and at home. The association between physical activity and health outcomes is frequently domain-dependent, and is captured by the term “physical activity paradox” [3]. For example, while leisure-time and transportation physical activity are consistently associated with positive health outcomes, occupational and household physical activity are often associated with risk factors for disease [3]. Previous studies suggest that occupational physical activity is the major contributor to total physical activity among people from low- and middle-income countries [4], reflecting social dynamics and status. The context of activity may change the effect of total physical activity on health outcomes.

In contrast to occupational and transportation physical activity, leisure-time physical activity is generally autonomous behavior, in which people can change their leisure-time behaviors if they choose and if they have opportunities to. But in most Latin American countries, prevalence of any leisure-time physical activity is less than 50% [5], indicating poor access to physical activity opportunities and facilities. In this region, physical activity, particularly during leisure-time, has increased over the years [5, 6] but inequalities related to income and gender also increased, and gender inequalities persist [5, 6].

Several Latin American countries have a national policy on physical activity [7] and there are consolidated programs to promote physical activity, such as the Ciclovia in Colombia, as well as the Agita São Paulo and the Academia da Cidade in Brazil [8]. Multiple school-based physical activity programs have increased physical activity levels [8]. But despite being promising, innovative, effective in their communities, and likely contributors to the general trend of increasing leisure-time physical activity in Latin America, these strategies insufficiently increased participation in physical activities and did not effectively reduce sociodemographic inequalities in participation.

Gender inequalities in physical activity practice are marked; from childhood, women have lower physical activity levels than men [2]. Gender cultural norms and the failure to adapt sports education and training methods for girls reduce their opportunities to engage in and enjoy leisure-time physical activity [9]. Women’s access to physical activity practices needs to be rethought. Beginning in childhood, they should be given more opportunities to practice a range of physical activity in school years and should have more access to exercise facilities, which requires changing cultural norms.

People of lower socioeconomic status should also be prioritized. Since they frequently live far from their jobs in cities and have less leisure time in which to be physical active, they may benefit if parks were built or revitalized in their communities. Most public physical activity facilities are located in wealthier neighborhoods [10]. To reduce inequity, interventions must account for unequal social and spatial distribution of physical activity initiatives in the urban environment, if they are to reach the neediest populations. Other social, economic, and political factors that create and sustain inequities must also be taken into account, since the problem in not just one of access.

There are other types of inequalities in physical activity practice. For example, older people and those with disability are less physically active than younger people and people without disabilities, indicating the necessity to incorporate physical activity into national health system. For instance, the Brazilian National Health Service (Sistema Único de Saúde in Portuguese) has addressed this by implementing the Family Health Strategy, which provides physical activity counselors.

There are many challenges to reducing inequalities in physical activity practice in Latin America, but addressing inequality should increase the level of physical activity at the population level and help prevent many non-communicable diseases. National physical activity policies must take action to promote physical activity in disadvantaged groups, including among women, people with limited financial resources, older adults, and people with disabilities.
